# Draft genome sequence data of two *Salmonella* bacteria from serogroup C type

**DOI:** 10.1016/j.dib.2020.105920

**Published:** 2020-06-22

**Authors:** Calvin Jiksing, Christopher Lok Yung Voo, Kenneth Francis Rodrigues

**Affiliations:** Biotechnology Research Institute, Universiti Malaysia Sabah, Jalan UMS, 88400, Kota Kinabalu, Sabah, Malaysia

**Keywords:** Salmonella, Genome, Whole genome sequencing, Comparative genomics

## Abstract

*Salmonella* is a gram-negative rod-shape bacterium from the family of *Enterobacteriaceae* that can cause a wide range of human disease such as enteric fever, gastroenteritis and bacteremia. Here we sequenced two genomes of *Salmonella* bacteria isolated from the *Gallus gallus domesticus* host. Genomic DNA of the two *Salmonella* isolates were extracted and subjected to whole genome sequencing using Illumina platform. The draft genome size of the two *Salmonella* isolates was determined to be 4,902,295 bp (S18) and 4,847,310 bp (S20) respectively. The percentage of GC content for both draft genomes is the same which is 52.1%. Both the whole genome shotgun project (S18 and S20) has been deposited in National Center for Biotechnology Information Sequence Read Archive under the accession number of SRR7503041 (S18) and SRR7503040 (S20). The sequenced genome (S18 and S20) were aligned with the reference genome and three other Salmonella genomes from serogroup B, D and E. The data obtained show the presence of unique DNA sequences in S18 and S20 genomes. This unique DNA sequences are from the fimbrial gene group.

**Specifications Table**

**Table:** 

**Subject**	Biology
**Specific subject area**	Bacteriology, Genomics
**Type of data**	Genomic sequence, gene annotation and comparative genomic of *Salmonella* serogroup C type.
**How data were acquired**	Whole genome was sequenced with an Illumina HiSeq System.
**Data format**	Raw sequencing reads, draft genome assembly, gene annotation and comparative genomics.
**Parameters for data collection**	*Salmonella* was isolated from dead chicken carcasses and cultured on Xylose-Lysine Deoxycholate (XLD) agar. Two genomes from serogroup C *Salmonella* was isolated using Wizard® Genomic DNA Purification kit and genus confirmation was conducted using the invasion (*invA*) gene.
**Description of data collection**	Raw read were trimmed using Trimmomatic software [1] and assembled using SPAdes [2]. Genome annotation was conducted using Rapid Annotation using Subsystem Technology (RAST) [3] (online web tool). Comparative genomic was done using CGView Comparison Tool (CCT) [4].
**Data source location**	*Salmonella* were isolated from dead chicken carcasses taken from few chicken farms in Sabah, Malaysia.
**Data accessibility**	The raw sequenced read was deposited at National Center for Biotechnology Information Sequence Read Archive database with accession number of SRR7503041 (S18) and SRR7503040 (S20). The deposited data can be accessed at **https://www.ncbi.nlm.nih.gov/sra/SRP152898**

## Value of the data

The comparative genomic data show the presence of serogroup-specific DNA fragment in the sequenced (S18 and S20) *Salmonella* genome
Data obtained can be utilized for identification of *Salmonella* serogroups using molecular method
These data can be used for the development of genetic marker in detecting *Salmonella* serogroup C type

## 1 Data

The data reported here are the genome sequencing, assembly, annotation and comparative genomic of two *Salmonella* isolates (S18 and S20) from serogroup C type. Raw reads from both genome were trimmed and assembled. The genome size for S18 is 4902,295 bp while the genome size for S20 is 4847,310 bp. The GC content for both genome are the same which is 52.1%. After genome assembly the S18 samples yielded 225 contigs while the S20 samples yielded 284 contigs. Both assembled genomes were uploaded to the Rapid Annotation using Subsystem Technology (RAST) server for annotation. Both draft genomes from S18 and S20 were then aligned with a reference genome and three other *Salmonella* genomes from serogroup B, D and E type using CGView Comparison Tool (CCT).

**Fig. 1:**
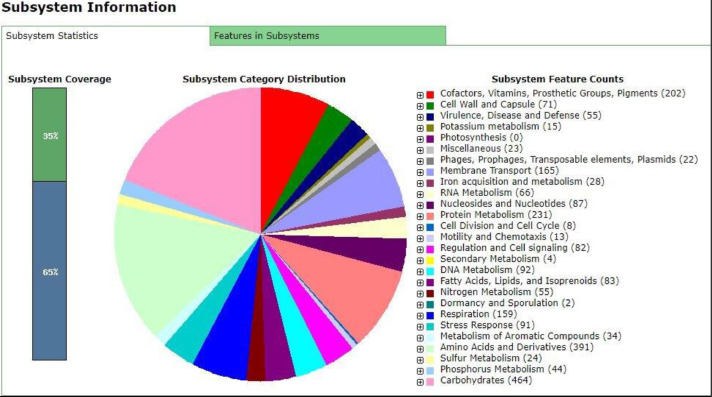
Subsystem distribution of the first draft genome (S18) based on the RAST annotation server.

**Fig. 2:**
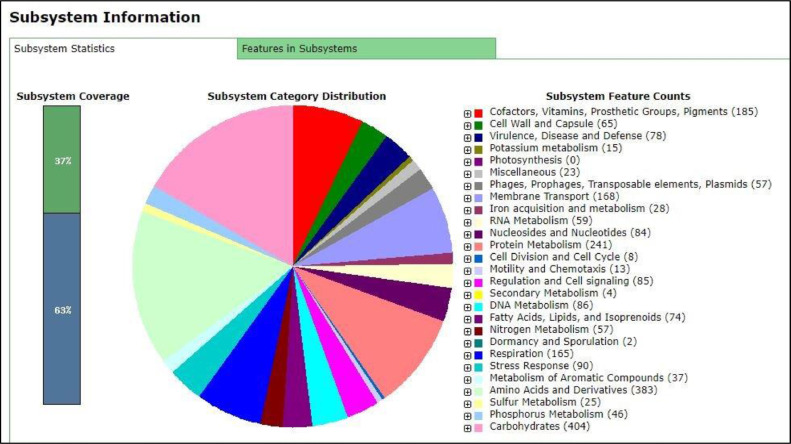
Subsystem distribution of the second draft genome (S20) based on the RAST annotation server.

**Fig. 3:**
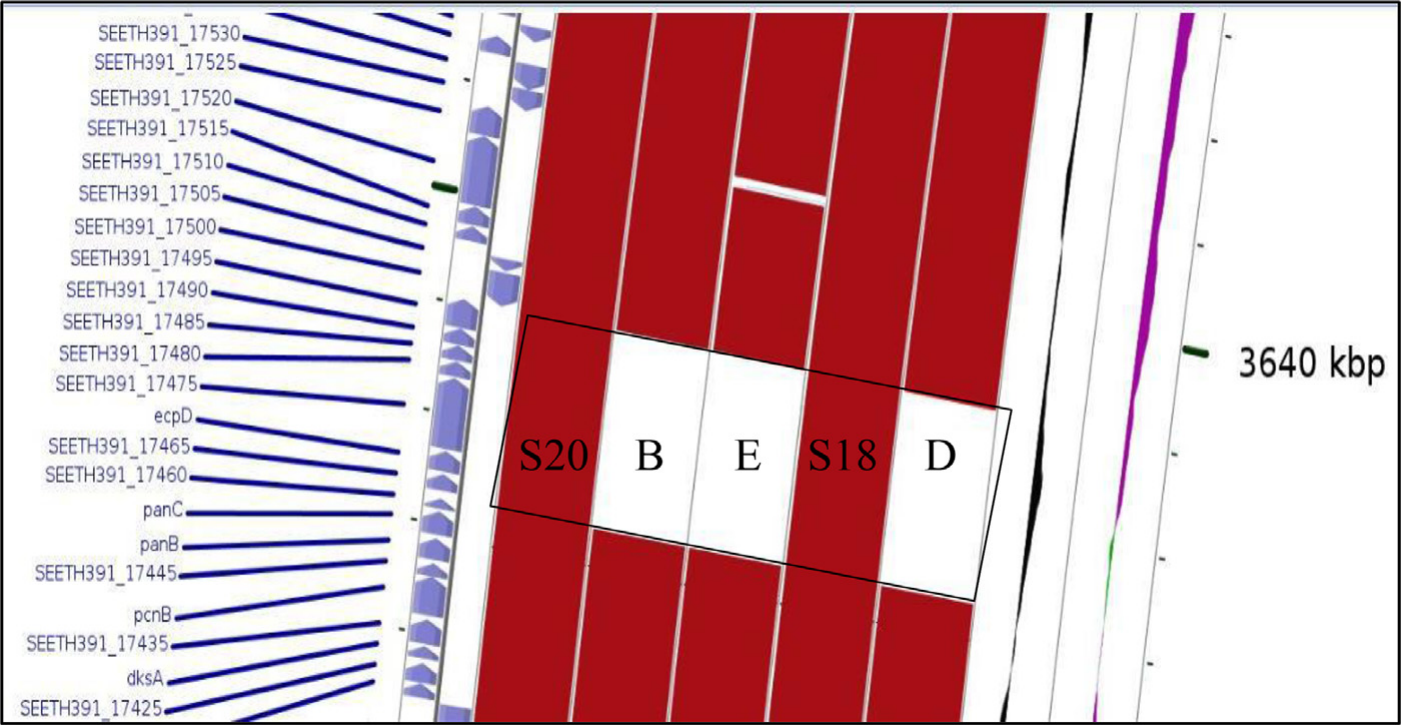
Comparative genomic analysis show the presence of unique DNA sequences in the sequenced genomes (S18 and S20) when compared with other *Salmonella* genomes from serogroup B, D and E. The white colour in the black boxes indicate the absence of this gene in *Salmonella* serogroup B, D and E genomes. S18 and S20 represent the sequenced genomes while alphabet B, D and E represent genomes from this serogroups type.

The genome annotation using Rapid Annotation using Subsystem Technology (RAST) server classified the S18 draft genome into 403 subsystems with 5005 numbers of coding sequences and 92 numbers of RNAs genes (Fig. 1). Meanwhile for S20 draft genome, Rapid Annotation using Subsystem Technology (RAST) server classified it into 404 subsystems with 4967 numbers of coding sequences and 79 numbers of RNAs genes (Fig. 2). The comparative genomic analysis meanwhile show the presence of serogroup-specific fragment in S18 and S20 draft genomes (Fig. 3) and this serogroup-specific fragments are originated from the fimbrial protein group. Primers were design to validate this data (Table 1).

**Table 1:** The table below show the number of positive sample (producing the expected band size) for each serogroup when tested with the designed primer. The alphabet B, C, D and E in the table below represent serogroup type. The “+” symbol represent presence of expected band while the “–” symbol represent no band produce or the band produce is other than the expected size. The number represent the sample number tested for each serogroup type.

	1	2	3	4	5	6	7	8	9	10	11	12	13	14	15	16
B	+	+	+	–	–											
C	+	+	+	+	+	+	+	+	+	+	+	+	+	+	+	–
D	–	–	–													
E	–	–	–	–	–	–										

## 2 Experimental design, materials, and methods

### 2.1 Genomic DNA extraction and sequencing

Prior to DNA extraction, *Salmonella* were serogroup following Jiksing et al. (2020) (in press) [5] method. Briefly, two *Salmonella* isolates (S18 and S20) were cultured overnight on the nutrient agar. These bacterial cultures were then serogrouped using the *Salmonella* Sero-Quick Group Kit (SSI Diagnostica, Denmark) following the manufacturer protocol. This kit comprised several type of antisera which is used to detect the antigen present on *Salmonella* surface using the slide agglutination method to identify the serogroup type. Two *Salmonella* isolates (S18 and S20) from the predominant serogroup (serogroup C) were then cultured overnight for genomes extraction. Genome extraction was conducted using Wizard® Genomic DNA Purification Kit following the manufacturer protocol. Library preparation using the New England Biolabs Next Ultra DNA Library Kit (350 bp insert) was conducted prior to sequencing process. The genomes was then sequenced using HiSeq 4000 sequencer (Illumina platform) for 150 paired-end reads.

### 2.2 Genome assembly, annotation and comparative genomic analysis

Raw reads for both genomes (S18 and S20) were trim with trimmomatic software prior to genome assembly. *De novo* assembly for both genomes (S18 and S20) were conducted using SPAdes software. The genome assembly quality was assessed using Quality Assessment Tool for Genome Assemblies (QUAST) software [6]. The contigs from both genomes were then annotated using Rapid Annotation using Subsystem Technology (RAST) server. Comparative genomic analysis for both genomes (S18 and S20) was conducted using the CGView Comparison Tool (CCT) software using the contigs produced in the genome assembly process. The sequenced genomes (S18 and S20) were aligned with a reference genome (GenBank Accession Number: CP011396.1) and three other genomes from serogroup B (GenBank Accession Number: CP019649.1), D (GenBank Accession Number: AM933172.1) and E (GenBank Accession Number: CP007531.1). These reference genome and three other genomes were retrieved from the National Center for Biotechnology Information (NCBI) databases.

## Declaration of Competing Interest

The authors declare that they have no known competing financial interests or personal relationships which have, or could be perceived to have, influenced the work reported in this article.

## Appendix Supplementary materials

Image, application 1
